# Establishment of a coculture system for *Porphyromonas gingivalis* and head and neck squamous cell carcinoma using spheroid culture and LATS inhibition

**DOI:** 10.1002/2211-5463.70154

**Published:** 2025-11-11

**Authors:** Yurika Nakajima, Shogo Okazaki, Muneaki Tamura, Shuichi Sato, Kenichi Imai

**Affiliations:** ^1^ Department of Microbiology and Immunology Nihon University School of Dentistry Tokyo Japan; ^2^ Department of Periodontology Nihon University School of Dentistry Tokyo Japan

**Keywords:** head and neck squamous cell carcinoma, Hippo pathway, *Porphyromonas gingivalis*, spheroid culture

## Abstract

*Porphyromonas gingivalis* (Pg) is a periodontal pathogen that has been implicated in the development and progression of head and neck squamous cell carcinoma (HNSCC). However, studying its interaction with HNSCC *in vitro* is challenging because of the obligatory anaerobic nature of Pg. To overcome this limitation, we developed a coculture system that enables the viability of both HNSCC cells and Pg using spheroid culture combined with Hippo pathway inhibition via treatment with a LATS1/2 inhibitor. In spheroid cultures, many HNSCC cell lines failed to grow in a normal medium. However, stable growth of these cells was achieved through Hippo pathway inhibition, which maintained the cells in an undifferentiated state. Furthermore, the addition of Pg to HNSCC spheroids maintained Pg viability in three out of four HNSCC cell lines, even after 3 days. Hippo pathway inhibition further enhanced Pg survival within the spheroids, likely by suppressing the differentiation‐induced expression of antimicrobial genes S100A8 and S100A9. Coculturing HNSCC cells with Pg did not promote spheroid growth but induced p38 activation, leading to increased expression of the proinflammatory cytokines IL‐1α and IL‐8. Database analysis using The Cancer Microbiome Atlas corroborated these findings, showing upregulation of p38 phosphorylation, IL‐1α, and IL‐8 in *Porphyromonas*‐positive HNSCC samples. These findings suggest that the established coculture system is a representative model of the clinical properties of Pg‐positive HNSCC and can serve as a valuable tool for investigating the long‐term interactions between HNSCC and viable Pg.

## Graphical abstract

We established a spheroid coculture system enabling viable *Porphyromonas gingivalis*–HNSCC interactions under normoxic conditions. Inhibition of LATS1/2 maintains tumor cells in an undifferentiated state, which may promote spheroid growth and create a more permissive environment for bacterial persistence. The model recapitulates features of *Porphyromonas*‐positive HNSCC, including p38‐mediated IL‐1α and IL‐8 induction, facilitating studies of bacterium‐driven tumor modulation.

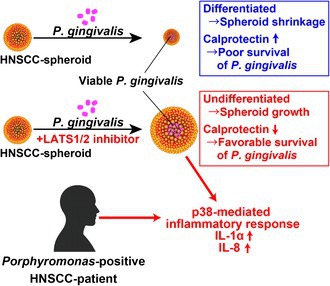

**Abbreviations**
HNSCC: head and neck squamous cell carcinoma
Pg: *Porphyromonas gingivalis*
TCGA: The Cancer Genome Atlas
TCMA: The Cancer Microbiome Atlas

The oral cavity contains abundant and diverse bacteria, which interact with the host, creating a unique microenvironment [1]. Recent studies have highlighted the role of microbial interactions in the development and progression of head and neck squamous cell carcinoma (HNSCC) [2, 3]. Notably, research that uses 4‐nitroquinoline‐1‐oxide induced HNSCC models has demonstrated that tumor‐associated microbiota can significantly accelerate carcinogenesis [4]. Moreover, periodontal disease, primarily driven by specific periodontal pathogens, has been associated with an increased risk of HNSCC development [5, 6, 7, 8]. Therefore, investigation of the mechanistic roles of these bacteria in HNSCC progression is urgently needed.

*Porphyromonas gingivalis* (Pg), a key Gram‐negative bacterium associated with periodontal disease, is significantly associated with the development and progression of HNSCC [6, 7]. Pg is frequently detected in HNSCC tissues and is associated with a high incidence of lymph node metastasis and poor prognosis [9, 10]. Studies in murine models have also demonstrated that Pg infection can accelerate tumor development and growth in HNSCC [11, 12].

However, because Pg is an obligate anaerobe that cannot survive under normoxic conditions, many studies investigating host cell–Pg interactions have relied on short‐term cocultures [12, 13], culture supernatants derived from Pg [14, 15, 16], or inactivated Pg [16, 17]. Although these studies yielded crucial insights, they failed to fully capture the interactions between viable Pg and HNSCC cells.

Previous studies demonstrated that the inner cores of multicellular spheroids can develop hypoxic conditions [18]. This characteristic of spheroids presents a potential opportunity for culturing obligate anaerobes, such as Pg, *in vitro*. However, a methodology for coculturing HNSCC cells with live Pg has not yet been established.

Activation of the Hippo pathway a well‐known tumor suppressor pathway, inhibits tumor development and growth in HNSCC [19, 20, 21, 22, 23, 24, 25, 26]. This pathway is activated by conditions such as loss of cell adhesion and increased cell density. Its activation triggers a cascade in which MST1/2 kinases phosphorylate and activate LATS1/2, leading to the degradation of the transcriptional coactivator YAP/TAZ. When not degraded, YAP/TAZ drives cell proliferation and promotes an undifferentiated or stem‐like state in HNSCC cells [27, 28].

This study aimed to develop an experimental system that allows for the coculture of viable Pg and HNSCC cells using spheroid culture. Inhibition of the Hippo pathway suppressed cellular differentiation and promoted spheroid growth, while downregulating the expression of antimicrobial genes such as S100A8 and S100A9. These changes may create a microenvironment favorable for the maintenance and proliferation of Pg within HNSCC spheroids. In addition, coculture with Pg did not enhance spheroid growth but induced the expression of proinflammatory cytokines IL‐1α and IL‐8 via p38 MAPK activation. Our findings indicate that the established coculture system may be a representative model of the clinical properties of Pg‐positive HNSCC.

## Materials and methods

### Cell lines

The HNSCC cell lines SAS, HSC‐2, HSC‐3, and HSC‐4 were obtained from RIKEN Cell Bank (Tsukuba, Ibaraki, Japan). The cells were cultured in Dulbecco's modified eagle medium supplemented with 10% fetal bovine serum and maintained at 37 °C and 5% CO_2_.

### Bacterial strains and culture

Pg strain ATCC 33277 (American Type Culture Collection, Manassas, VA, USA) was cultured in Gifu Anaerobic Broth, Modified supplemented with 5 μg·mL^−1^ hemin and 0.5 μg·mL^−1^ menadione under anaerobic conditions.

To examine the regrowth of Pg from the spheroids under these conditions, each cocultured spheroid was placed in separate tubes and incubated for 3 day at 37 °C under anaerobic conditions. When a bacterial suspension was observed in the culture medium, a portion of the suspension was collected for DNA extraction. The extracted DNA was then subjected to PCR using Pg‐specific 16S rRNA gene primers to confirm the presence of Pg. In parallel, the remaining suspension was spread on a blood agar plate and subsequently cultured for 4 day under anaerobic conditions.

The primer sequences used for PCR were as follows (5′ to 3′): Pg 16S rRNA, AGGCAGCTTGCCATACTGCG, and ACTGTTAGCAACTACCGATGT.

### Spheroid culture and treatment

Spheroids were cultured using an EZ‐BindShut®II 96‐well round‐bottom plate (4870‐800LP; Iwaki, Japan). Cells were seeded into the 96‐well plate at a density of 10 000 cells/well and centrifuged at 1000 ***g*** for 5 min at room temperature. The cells were incubated at 37 °C for 1 day to facilitate spheroid formation. Subsequently, LATS‐IN‐1 (1 μm; Cayman Chemical, Ann Arbor, MI, USA), verteporfin (2 μm; Cayman Chemical), Pg (10^7^ colony‐forming unit·mL^−1^), or the corresponding solvent was added to the spheroids. Spheroid size was measured daily from Days 1 to 4, and spheroid size was assessed using the fiji software [29].

### Quantitative reverse transcription PCR (RT‐PCR)

RNA was extracted from HNSCC cells using the ReliaPrep RNA Cell Miniprep System (Promega, Madison, WI, USA). For the extraction of RNA from the spheroids, at least five spheroids per sample were used. Subsequently, cDNA was synthesized using a ReverTra Ace qPCR RT Master Mix (Toyobo, Osaka, Japan), and this cDNA mixture was subjected to real‐time PCR analysis on a Thermal Cycler DICE Real‐Time System (TP951; Takara Bio, Shiga, Japan) using KOD SYBR qPCR Mix (Toyobo). The primer sequences used are as follows (5′ to 3′):

*CA9*: GTGCCTATGAGCAGTTGCTGTC/AAGTAGCGGCTGAAGTCAGAGG

*VEGFA*: TTGCCTTGCTGCTCTACCTCCA/GATGGCAGTAGCTGCGCTGATA

*CTGF*: CAGCATGGACGTTCGTCTG/AACCACGGTTTGGTCCTTGG

*ANKRD1*: AGTAGAGGAACTGGTCACTGG/TGTTTCTCGCTTTTCCACTGTT

*S100A8*: ATGCCGTCTACAGGGATGACCT/AGAATGAGGAACTCCTGGAAGTTA

*S100A9*: GCACCCAGACACCCTGAACCA/TGTGTCCAGGTCCTCCATGATG

*IL1A*: TGTATGTGACTGCCCAAGATGAAG/AGAGGAGGTTGGTCTCACTACC

*IL8*: GAGAGTGATTGAGAGTGGACCAC/CACAACCCTCTGCACCCAGTTT

*RPS1*7: AGGAGATCGCCATTATCCCCA/GTTGGACAGACTGCCGAAGT

*RPS17* was used as an endogenous control for the normalization of gene expression levels.

### Western blotting

At least five spheroids per sample were collected. Whole cell lysates were prepared by directly lysing spheroids in 1× Laemmli buffer. Nuclear fractions were extracted using the NE‐PER™ Nuclear and Cytoplasmic Extraction Reagents (Thermo Fisher Scientific, Waltham, MA, USA) according to the manufacturer's protocol and mixed with 4× Laemmli buffer. All samples were then boiled with 10 mm dithiothreitol and subjected to sodium dodecyl sulfate/polyacrylamide gel electrophoresis. The separated proteins were then transferred onto polyvinylidene difluoride membranes, which were blocked using 5% skim milk and incubated with anti‐YAP (#4912; Cell Signaling Technology; Danvers, MA, USA), anti‐IκB (#4814; Cell Signaling Technology), anti‐NFκB (#4764; Cell Signaling Technology), anti‐phosphorylated NFκB (#3033; Cell Signaling Technology), anti‐p38 (#8690; Cell Signaling Technology), anti‐phosphorylated p38 (#4511; Cell Signaling Technology), or anti‐β‐actin (sc‐47 778; Santa Cruz Biotechnology, Santa Cruz, CA, USA) primary antibodies. Subsequently, the membranes were washed with Tris‐buffered saline containing Tween 20 and incubated with horseradish peroxidase‐labeled anti‐rabbit (111‐035‐144; Jackson ImmunoResearch Laboratories, West Grove, PA, USA) or anti‐mouse (NA931V; Cytiva, Tokyo, Japan) secondary antibodies. After washing, the immune complexes were detected using SuperSignal West Pico PLUS Chemiluminescent Substrate (Thermo Fisher Scientific).

### Quantification of Pg per spheroid

To quantify the number of Pg in the spheroids, we lysed a single spheroid and a stepwise diluted Pg suspension of a known density using 100 μL of DNA extraction buffer supplemented with 1 μL of proteinase K (Promega) at 55 °C for 30 min. DNA was purified via phenol‐chloroform extraction, followed by ethanol precipitation. Finally, we performed quantitative PCR (qPCR) targeting the 16S rRNA gene of Pg. Calibration curves were constructed using *C*_t_ values obtained from the known densities of Pg. The number of bacterial cells in each spheroid was calculated. The same primer set used in the conventional PCR analysis of Pg 16S rRNA was applied for qPCR.

### TCGA analysis

HNSCC patient data from the TCGA dataset were categorized into *Porphyromonas*‐positive and ‐negative groups based on the detection of *Porphyromonas* in TCMA data. Gene expression was analyzed using RNA sequencing data. The levels of phosphorylated p38 and phosphorylated NF‐κB were quantified using reverse‐phase protein array (RPPA) data normalized using replicate‐based normalization. RNA sequencing and RPPA data of patients with HNSCC were downloaded from UCSC Xena (https://xena.ucsc.edu/) [30]. The abundance data for *Porphyromonas* in TCGA datasets were obtained from TCMA (https://tcma.pratt.duke.edu/) [31].

### Statistical analysis

All data are presented as the mean ± standard error. Statistical analyses were conducted using Student's *t*‐test for comparisons between two groups and one‐way analysis of variance followed by Tukey's honest significant difference test for multiple‐group comparisons, using Prism 10 (version 10.1.10).

## Results

### Most HNSCC cell lines exhibit limited proliferation under spheroid culture conditions

**Fig. 1:**
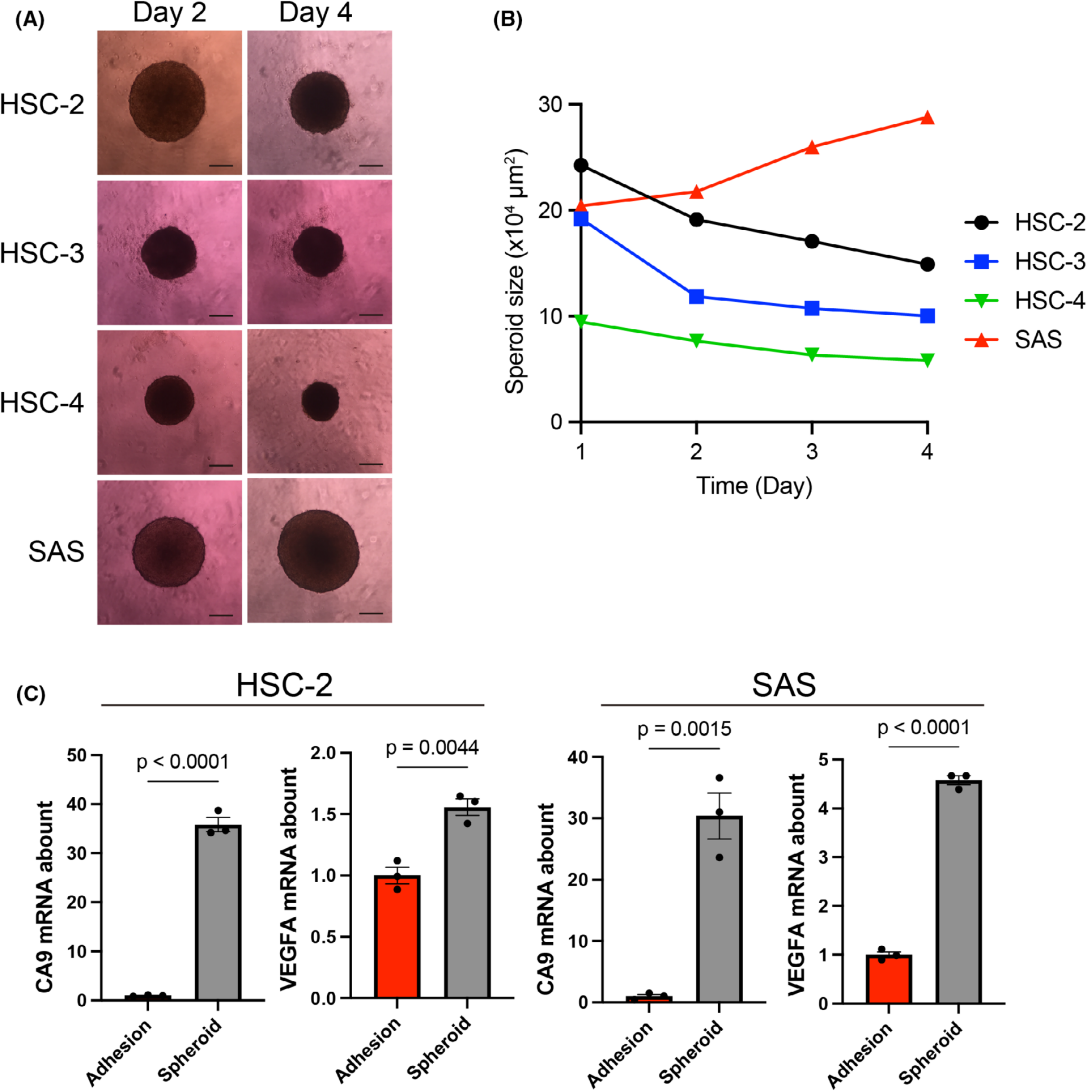
Spheroid growth of HNSCC cell lines. (A) Phase contrast images of spheroids derived from HSC‐2, HSC‐3, HSC‐4, and SAS cells on days 2 and 4. Scale bar, 200 μm. (B) Changes in spheroid size of HSC‐2, HSC‐3, HSC‐4, and SAS from days 1 to 4. Data are expressed as the mean ± SE of five independent experiments. (C) Quantitative RT‐PCR analysis of *CA9* and *VEGFA* expression in HSC‐2 and SAS cells after 2 days under adhesion and spheroid culture conditions. Data are expressed as the mean ± SE of three independent experiments. Statistical significance was determined using an unpaired two‐tailed Student's *t*‐test.

Prior to the establishment of a coculture system of HNSCC cells and Pg, we evaluated the proliferative capacity of HSC‐2, HSC‐3, HSC‐4, and SAS cells in spheroid cultures without Pg. Monitoring of spheroid size over time revealed a notable increase in SAS spheroid size, whereas HSC‐2, HSC‐3, and HSC‐4 spheroids progressively decreased in size (Fig. 1A,B). Consistent with previous reports showing that only three of 11 HNSCC cell lines form proliferative spheroids, whereas the others remain dormant or undergo cell death under spheroid conditions [32], our results indicate that most HNSCC cell lines exhibit limited proliferative capacity in spheroid culture.

To further characterize the spheroid environment, we examined the mRNA expression of the hypoxia‐responsive genes *CA9* and *VEGFA* (Fig. 1C). As expected, spheroid culture significantly upregulated *CA9* and *VEGFA* expression in both HSC‐2 and SAS cells, indicating the induction of hypoxic conditions during spheroid formation. These findings suggest that HNSCC spheroids provide a permissive environment for anaerobic coculture. Therefore, we aimed to establish a method that enables the stable spheroid growth of HNSCC cells.

### The Hippo pathway is activated in HNSCC cells that cannot grow under spheroid culture conditions

**Fig. 2:**
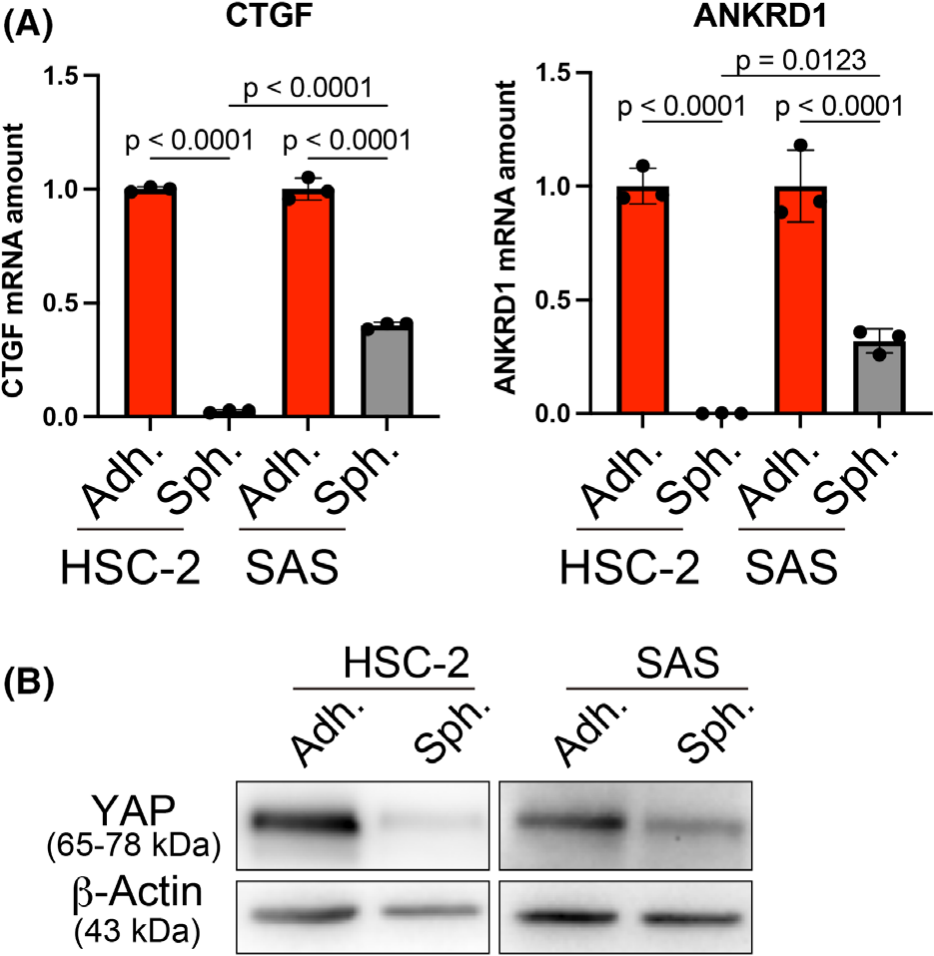
Spheroid culture activates the Hippo pathway in HNSCC cells. (A) Quantitative RT‐PCR analysis of *CTGF* expression in HSC‐2 and SAS cells after 2 days under adhesion and spheroid culture conditions. Data are expressed as the mean ± SE of three independent experiments. Statistical significance was determined using a one‐way ANOVA with Tukey's *post hoc* test. (B) Immunoblot analysis of the protein expression of YAP and β‐Actin (loading control) in HSC‐2 and SAS cells cultured under adhesion and spheroid conditions for 2 days.

Given that the Hippo pathway is activated by the loss of adhesion, we hypothesized that its activation is involved in the inhibition of HNSCC spheroid growth. We examined Hippo pathway activity in proliferative SAS and nonproliferative HSC‐2 cells under spheroid culture conditions, as shown in Fig. 1. Expression of *CTGF* and *ANKRD1*, which are suppressed by activation of the Hippo pathway, was examined in these cells under adhesion and spheroid culture conditions. The results revealed that *CTGF* and *ANKRD1* were downregulated in both HSC‐2 and SAS cells under spheroid culture compared with adherent conditions; however, the decrease was more significant in HSC‐2 cells (Fig. 2A). In addition, the protein expression of YAP, a transcriptional coactivator that is degraded through the activation of the Hippo pathway, markedly decreased in HSC‐2 cells compared with SAS cells upon spheroid culture (Fig. 2B). These results demonstrate that spheroid culture activates the Hippo pathway in HNSCC cells that cannot grow under spheroid culture conditions.

### Hippo pathway inhibition induces HNSCC cell growth under spheroid culture conditions

**Fig. 3:**
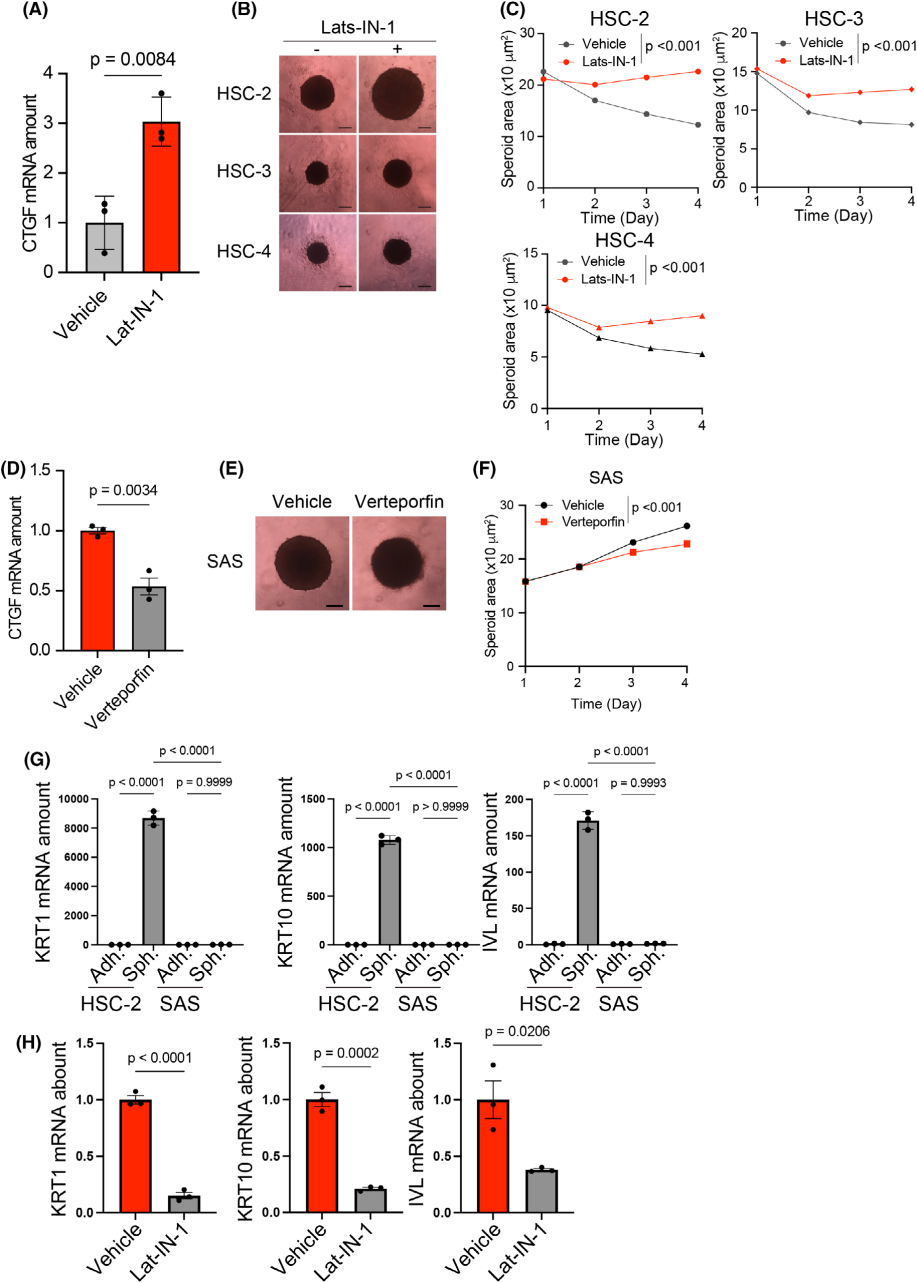
Hippo pathway inhibition induces spheroid growth of HNSCC cells. (A) Quantitative RT‐PCR analysis of *CTGF* expression in spheroid cultures of HSC‐2 cells in the absence or presence of LATS‐IN‐1 for 3 days. Data were normalized to the *RPS17* mRNA level and are expressed as the mean ± SE. Statistical significance was determined using an unpaired two‐tailed Student's *t*‐test. (B) Phase contrast images of spheroids derived from HSC‐2, HSC‐3, and HSC‐4 cells on day 4 in the absence or presence of LATS‐IN‐1. (C) Changes in spheroid size of HSC‐2, HSC‐3, and HSC‐4 from days 1 to 4 in the absence or presence of LATS‐IN‐1. (D) Quantitative RT‐PCR analysis of *CTGF* expression in spheroid cultures of SAS cells in the absence or presence of verteporfin for 1 day. Data were normalized to the *RPS17* mRNA level and are expressed as the mean ± SE. Statistical significance was determined using an unpaired two‐tailed Student's *t*‐test. (E) Phase contrast images of spheroids derived from SAS cells on day 4 in the absence or presence of verteporfin. (F) Changes in spheroid size of SAS from days 1 to 4 in the absence or presence of verteporfin. (G) Quantitative RT‐PCR analysis of *KRT1, KRT10*, and *IVL* expression in spheroid cultures of HSC‐2 and SAS cells under adhesion and spheroid culture for 2 days. Data were normalized to the *RPS17* mRNA level and are expressed as the mean ± SE. Statistical significance was determined using a one‐way ANOVA with Tukey's *post hoc* test. (H) Quantitative RT‐PCR analysis of *KRT1*, *KRT10*, and *IVL* expression in spheroid cultures of HSC‐2 cells in the absence or presence of LATS‐IN‐1 for 3 days. Data were normalized to the *RPS17* mRNA level and are expressed as the mean ± SE. Statistical significance was determined using an unpaired two‐tailed Student's *t*‐test.

Next, we investigated whether LATS‐IN‐1, also known as TRULI, an inhibitor of LATS1/2 [33, 34], induced cell proliferation in spheroid cultures. Treatment of HSC‐2 spheroids with LATS‐IN‐1 induced *CTGF* expression, confirming the inhibition of the Hippo pathway (Fig. 3A). In HSC‐2, HSC‐3, and HSC‐4 cells, which failed to proliferate in spheroid culture, LATS‐IN‐1 treatment induced spheroid growth (Fig. 3B,C). To further examine the functional involvement of YAP activity in spheroid growth, we treated SAS spheroids with verteporfin, an inhibitor of the YAP‐TEAD interaction. *CTGF* expression was significantly downregulated following verteporfin treatment (Fig. 3D), confirming YAP inhibition. In addition, verteporfin suppressed spheroid growth in SAS cells (Fig. 3E,F), and at higher concentrations, it led to spheroid disintegration (data not shown). To explore the mechanism through which Hippo pathway inhibition promotes spheroid growth, we focused on the differentiation status of HNSCC cells, based on previous reports that adhesion restriction can induce differentiation [35, 36], and that YAP activity is involved in maintaining an undifferentiated state [27, 28]. In nonproliferative HSC‐2 cells, the expression of differentiation markers *KRT1*, *KRT10*, and *IVL* was markedly upregulated under spheroid culture conditions, indicating induction of differentiation. In contrast, proliferative SAS cells showed no such upregulation, revealing that they retained an undifferentiated phenotype in spheroid culture (Fig. 3G). Notably, treatment with LATS‐IN‐1 suppressed the induction of these differentiation markers in HSC‐2 cells (Fig. 3H). These findings indicate that Hippo pathway activation suppresses HNSCC cell proliferation in spheroid culture, whereas its inhibition by LATS‐IN‐1 promotes stable spheroid growth, at least in part by preventing cellular differentiation.

### Spheroid culture enables the coculture of Pg and HNSCC cells under normoxic conditions

**Fig. 4:**
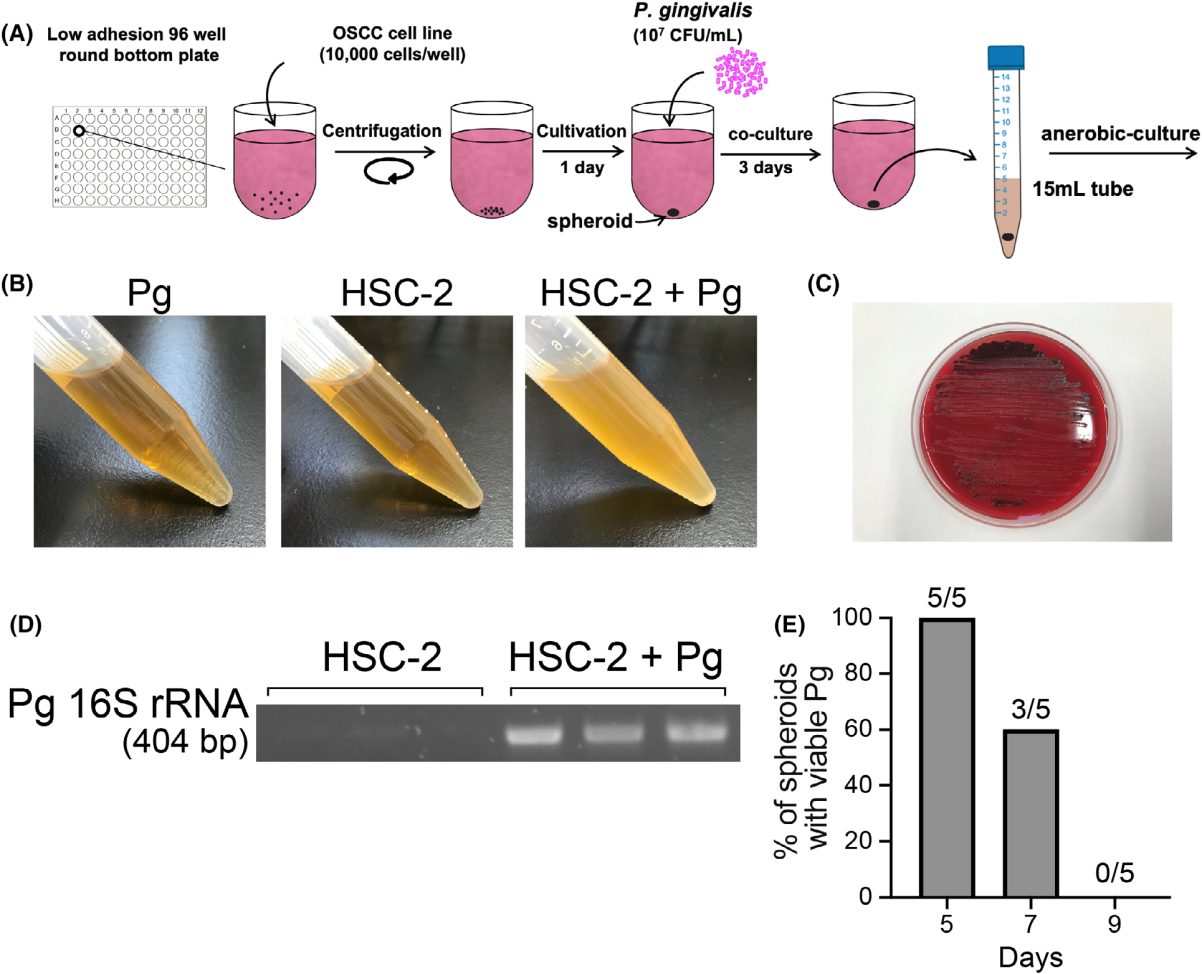
Spheroid culture enables the long‐term coculture of Pg and HNSCC cells. (A) Schema for coculture of HNSCC cells and Pg using spheroid culture and anaerobic reculturing of Pg. (B) Medium turbidity after culturing Pg or HSC‐2 alone or co‐culturing HSC‐2 cells and Pg for 3 days, followed by 3 days of anaerobic culture. (C) Black‐pigmented colonies formed on blood agar plates due to bacterial growth as in (B). (D) PCR analysis of DNA extracted from the bacterial suspensions shown in (B) using Pg‐specific 16S rRNA primers. (E) Percentage of spheroids from which viable Pg regrew after 3 days of anaerobic culture following coculture for the indicated number of days. Data indicate the number of spheroids with regrowth out of five analyzed.

Next, we examined whether the spheroid culture enabled the coculture of HNSCC cells and Pg under normoxic conditions. Pg was added 1 day after the initiation of the spheroid culture of HNSCC cells, and the cells were cultured in the presence or absence of LATS‐IN‐1 for 3 days. Subsequently, Pg was recultured under anaerobic conditions to evaluate its survival (Fig. 4A). As shown in Fig. 4B, no medium turbidity resulting from bacterial growth was observed in the recultures with Pg or HSC‐2 spheroids alone. However, the addition of Pg to HSC‐2 spheroids resulted in medium turbidity (Fig. 4B). Furthermore, the formation of black‐pigmented colonies on blood agar, a characteristic feature of Pg, together with the detection of a clear PCR band using Pg‐specific primers, confirmed that the proliferating bacteria in the coculture were indeed Pg (Fig. 4C,D).

We then examined whether the coculture of HNSCC cells and Pg in spheroid culture was possible in several cell lines, both in the absence and in the presence of LATS‐IN‐1. The results showed that Pg could be cocultured with HNSCC cells, except for HSC‐3 cells, in the presence or absence of LATS‐IN‐1 (Table 1).

**Table 1:** Success rate of establishing cocultures of HNSCC cells and Pg. The success rate of establishing cocultures of HNSCC cells and Pg was evaluated in the absence and presence of LATS‐IN‐1. Data are expressed as the number of regrowths in the anaerobic culture per number of spheroids. NT, not tested.

LATS‐IN‐1	SAS	HSC‐2	HSC‐3	HSC‐4
−	3/3	3/3	0/3	3/3
+	NT	3/3	0/3	3/3

Finally, we evaluated the duration for which viable Pg could be maintained within the spheroids. On Day 5, viable Pg was detected in all five spheroids examined (100%), but this decreased to 60% by Day 7 and 0% by Day 9 (Fig. 4E). These results indicate that this coculture system allows stable maintenance of viable Pg within HNSCC spheroids for up to approximately 5 days.

### Hippo pathway inhibition enhances Pg survival in spheroids, possibly through suppression of S100A8/9 expression

**Fig. 5:**
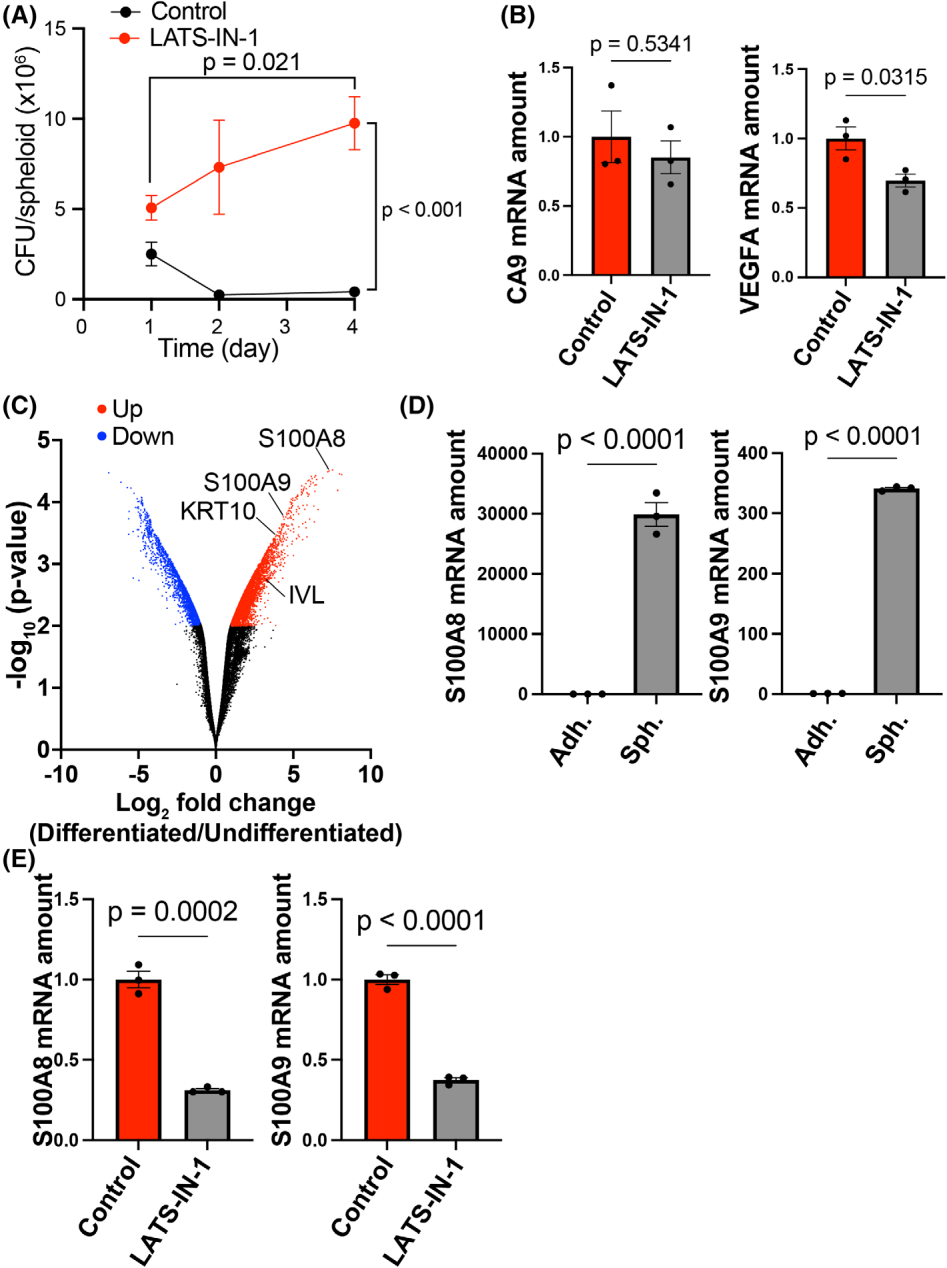
LATS inhibition promotes Pg survival and modulates antimicrobial gene expression in HSC‐2 spheroid cultures. (A) DNA was extracted from a single spheroid of HSC‐2 and Pg cocultures, and the number of bacteria per spheroid was determined using quantitative PCR against the Pg 16S rDNA sequence on days 1, 2, and 4. Data are expressed as the mean ± SE of three independent experiments. (B) Quantitative RT‐PCR analysis of *CA9* and *VEGFA* expression in spheroid cultures of HSC‐2 cells in the absence or presence of LATS‐IN‐1 for 3 days. Data were normalized to the *RPS17* mRNA level and are expressed as the mean ± SE. Statistical significance was determined using an unpaired two‐tailed Student's *t*‐test. (C) Volcano plot of microarray analysis comparing differentiated and undifferentiated HSC‐2 cells. Upregulated genes in the differentiated state (right side) and in the undifferentiated state (left side) are shown in red and blue, respectively. Antimicrobial factors S100A8 and S100A9, as well as differentiation markers KRT10 and IVL, are indicated. (D) Quantitative RT‐PCR analysis of *S100A8* and *S100A9* expression in spheroid cultures of HSC‐2 cells under adhesion and spheroid culture condition for 2 days. Data were normalized to the *RPS17* mRNA level and are expressed as the mean ± SE. Statistical significance was determined using an unpaired two‐tailed Student's *t*‐test. (E) Quantitative RT‐PCR analysis of *S100A8* and *S100A9* expression in spheroid cultures of HSC‐2 cells in the absence or presence of LATS‐IN‐1 for 3 d. Data were normalized to the *RPS17* mRNA level and are expressed as the mean ± SE. Statistical significance was determined using an unpaired two‐tailed Student's *t*‐test.

To evaluate the effect of LATS‐IN‐1 on the coculture of HNSCC cells and Pg, the amount of Pg in the spheroids was evaluated over time. In the absence of LATS‐IN‐1, Pg content in the spheroids rapidly decreased by Day 2, with only a small amount of surviving Pg. In contrast, treatment of spheroids with LATS‐IN‐1 prevented this decrease, and Pg proliferated within the spheroids from Days 1 to 4 (Fig. 5A).

Next, we investigated the mechanism by which LATS‐IN‐1 enhanced the efficiency of Pg coculture. Because LATS‐IN‐1 increased spheroid size, we hypothesized that the expansion of hypoxic regions within the spheroid might contribute to improved Pg maintenance. However, LATS‐IN‐1 treatment did not increase the expression of hypoxia‐responsive genes *CA9* and *VEGFA*, indicating that hypoxia was not further enhanced (Fig. 5B).

As LATS‐IN‐1 suppresses differentiation, and previous studies have reported that keratinocyte differentiation alters the expression of antimicrobial molecules, we considered the possibility that LATS‐IN‐1 promotes Pg coculture by suppressing such antimicrobial responses [37, 38, 39]. Our previously reported microarray analysis of HSC‐2 cells revealed that in addition to differentiation markers, the expression of *S100A8* and *S100A9* was upregulated under differentiation‐inducing conditions (Fig. 5C). Because S100A8 and S100A9 form a heterodimer known as calprotectin, which exhibits antimicrobial activity against various bacteria including Pg, we further examined whether their expression was modulated by LATS‐IN‐1 treatment [40, 41]. As expected, spheroid culture markedly upregulated *S100A8* and *S100A9* expression compared with that in adherent culture (Fig. 5D), whereas treatment with LATS‐IN‐1 suppressed this upregulation in spheroid culture (Fig. 5E).

These findings indicate that inhibition of the Hippo pathway suppresses the expression of antimicrobial molecules such as S100A8 and S100A9 by blocking differentiation, thereby facilitating the maintenance and proliferation of Pg within HNSCC spheroids.

### Coculture of Pg and HNSCC cells induces cytokine expression via p38 MAPK signaling activation

**Fig. 6:**
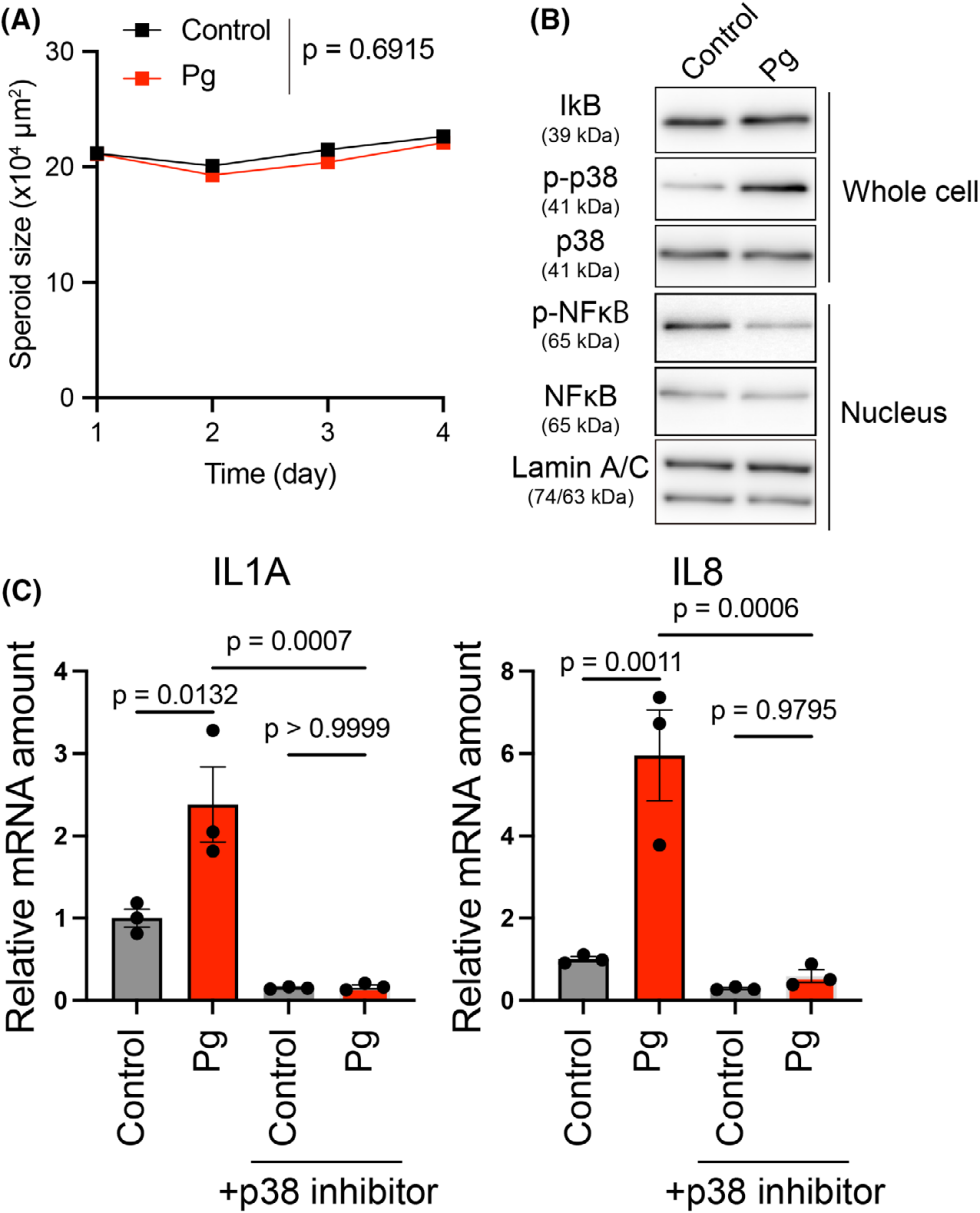
p38‐dependent expression of proinflammatory cytokines by coculture of HNSCC cells and Pg. (A) Changes in spheroid size of HSC‐2 cocultured with or without Pg in the presence of LATS‐IN‐1. Spheroid growth was monitored until day 9. Statistical comparison was performed on day 4, when Pg was confirmed to stably survive within the spheroids. Data are expressed as the mean ± SE. (B) Immunoblot analysis was performed using whole‐cell lysates to detect IκB and phosphorylated p38, and nuclear extracts to detect NF‐κB and phosphorylated NF‐κB. Total p38 and Lamin A/C served as loading controls for whole‐cell and nuclear fractions, respectively. (C) Quantitative RT‐PCR analysis of *IL1A* and *IL8* expression in HSC‐2 spheroids cocultured with or without Pg in the absence or presence of a p38 inhibitor. Data are expressed as the mean ± SE. Statistical significance was determined using a one‐way ANOVA with Tukey's *post hoc* test.

To investigate the effects of Pg on HNSCC cells within our established coculture system, we first assessed spheroid growth in the presence of Pg. As shown in Fig. 6A, the addition of Pg did not significantly affect the growth of the HNSCC spheroids compared to spheroids without Pg. This suggests that Pg does not directly influence the proliferation of HNSCC cells under these experimental conditions.

Next, we examined the effect of Pg on inflammation‐related signals, NF‐kB and p38 MAPK, which have been reported to be activated by Pg [42, 43, 44]. We assessed both total and phosphorylated NF‐κB in nuclear fractions; however, no apparent upregulation was observed. In addition, the expression of IκB in whole cell lysates remained unchanged. These findings indicate that NF‐κB signaling was not activated under these conditions. However, we observed an increase in p38 phosphorylation, suggesting activation of the p38 MAPK pathway (Fig. 6B). The expression of inflammatory cytokines regulated by p38 was also examined, and coculture with Pg was found to induce the expression of IL‐1α and IL‐8, which was suppressed by a p38 inhibitor (Fig. 6C). These results demonstrate that coculture of HNSCC cells with Pg induces inflammatory cytokine expression through p38 activation.

### Pg‐HNSCC coculturing system recapitulates the characteristics observed in clinical specimens

**Fig. 7:**
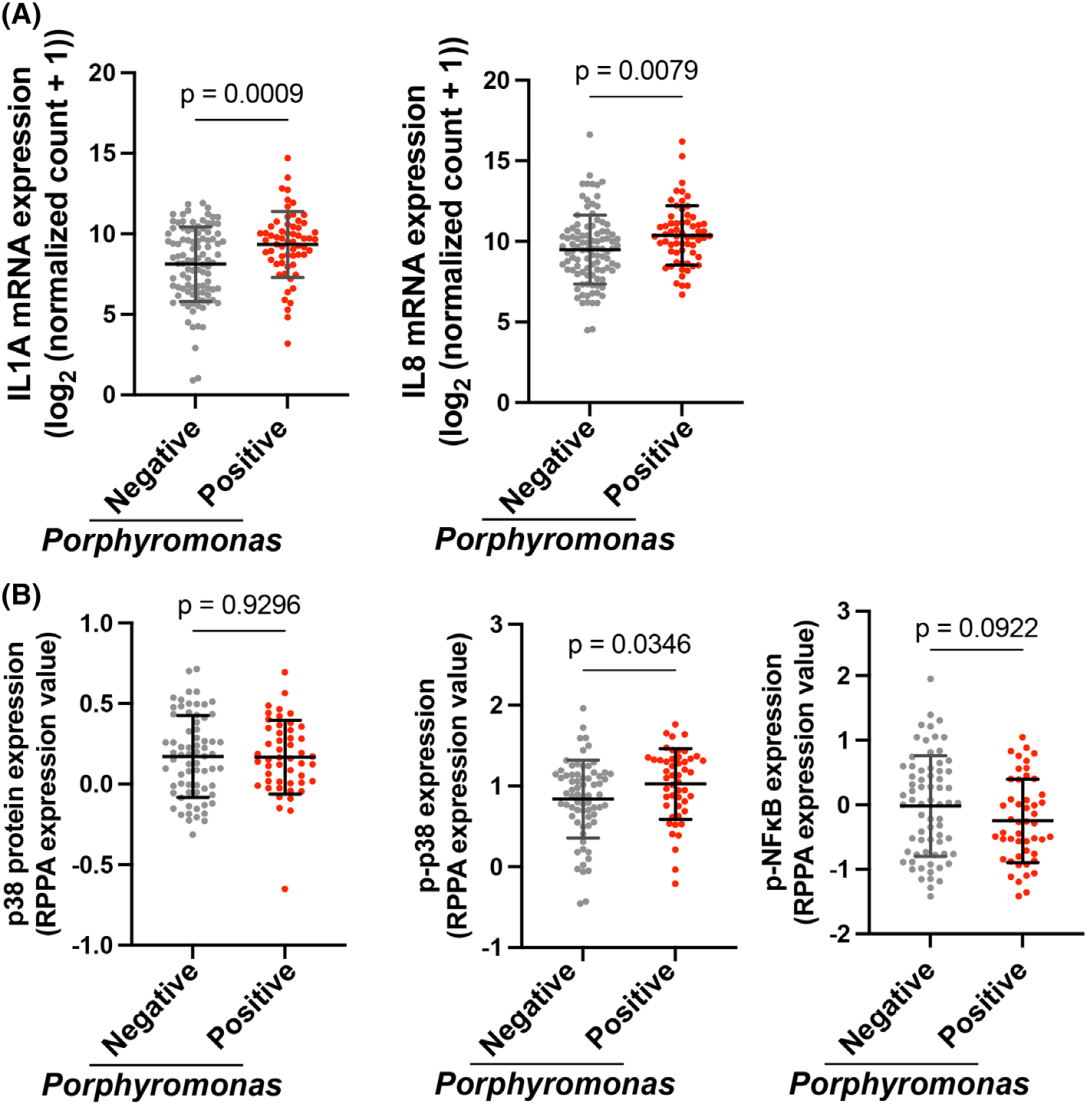
Inflammatory cytokine expression and p38 phosphorylation in *Porphyromonas*‐positive and ‐negative HNSCC clinical samples. mRNA expression of (A) *IL1A* (left) and *IL8* (right) in *Porphyromonas*‐positive and ‐negative HNSCC samples. Statistical significance was determined using an unpaired two‐tailed Student's *t*‐test. (B) Expression level of total p38 (left), phosphorylated p38 (right) and phosphorylated NF‐κB in *Porphyromonas*‐positive and ‐negative HNSCC samples. Statistical significance was determined using an unpaired two‐tailed Student's *t*‐test.

To examine whether the effect of Pg on HNSCC observed in our coculture system reflects responsiveness to Pg in patient‐derived HNSCC, we analyzed The Cancer Genome Atlas (TCGA) HNSC dataset and its microbiome profile from TCGA, which consists of microbiome data derived from TCGA samples [31, 45]. A comparison of cytokine expression between *Porphyromonas*‐positive and ‐negative groups showed that both IL‐1A and IL‐8 were highly expressed in the *Porphyromonas*‐positive group, consistent with our coculture system (Fig. 7A). We also examined the expression of p38 and found that its phosphorylation was higher in the *Porphyromonas*‐positive group, whereas total p38 expression did not differ. In contrast, phosphorylated NF‐κB levels showed no apparent difference between the two groups. These observations are consistent with our coculture experiments, in which Pg‐induced p38 activation but did not promote NF‐κB phosphorylation (Fig. 7B). These results indicate that our Pg‐HNSCC coculture system recapitulates the characteristics observed in clinical HNSCC specimens.

## Discussion

In this study, we established an experimental system in which HNSCC cells could be cocultured with an obligate anaerobic bacterium, Pg, under normoxic conditions, using spheroid culture and the Hippo pathway inhibitor LATS‐IN‐1. Although coculture systems using spheroids with other bacterial species such as *Fusobacterium nucleatum* and *Salmonella typhimurium* have been previously reported in colorectal and breast cancers [46, 47, 48], this is the first report of a coculture system capable of long‐term cultivation of viable bacteria with HNSCC cells. This system provides a platform for analyzing the interactions between viable Pg and HNSCC cells *in vitro*.

Under spheroid culture conditions, many HNSCC cell lines failed to grow and exhibited reduced YAP activity. Interestingly, the addition of LATS‐IN‐1, a recently developed inhibitor of the Hippo pathway activator LATS1/2 [33, 34], enabled stable spheroid growth across multiple HNSCC cell lines, likely through the suppression of cellular differentiation. Our findings suggest that inactivation of YAP/TAZ via the Hippo pathway is critical for growth inhibition in spheroid cultures. While YAP/TAZ activation is frequently observed in HNSCC and is known to drive tumor progression [19, 49, 50], our findings indicate that this activation is not solely governed by intrinsic signaling and exogenous factors involved in YAP/TAZ activation. Therefore, these exogenous factors that activate YAP/TAZ may represent promising therapeutic targets for YAP/TAZ‐driven tumor growth.

In cocultures of HNSCC cells and Pg, Pg was maintained in many HNSCC cell lines with or without LATS1/2 inhibitors. However, coculture was not possible in HSC‐3 cells. Infection of host tissues with Pg requires adhesion, invasion, and survival in the presence of host‐derived reactive oxygen species and other defense factors [51]. Pg infection may be inhibited at any of these stages in HSC‐3 cells. Potential explanations include inherent differences in cell surface receptors involved in bacterial adhesion or invasion, higher basal expression of antimicrobial peptides, or metabolic properties that render the spheroid microenvironment less permissive to Pg colonization. Further studies may help elucidate the factors that influence Pg infection and survival. Although the addition of LATS‐IN‐1 did not impact the success rate of establishing a Pg coculture, it effectively suppressed the decline in Pg content and promoted Pg proliferation within spheroids. These findings suggest that LATS‐IN‐1 may modify the microenvironment to facilitate Pg growth, possibly by suppressing differentiation‐induced expression of antimicrobial factors such as *S100A8* and *S100A9*. Further detailed studies are necessary to fully elucidate the underlying mechanisms driving these effects. Additionally, this system enabled the quantification of Pg within individual spheroids, providing a valuable tool for studying the factors and testing drugs that influence Pg survival and proliferation in infected cells.

Our analysis revealed that coculture with Pg did not enhance spheroid growth but induced the expression of proinflammatory cytokines IL‐1α and IL‐8 via p38 MAPK activation. In addition, our analysis of HNSCC clinical sample data from TCGA and TCMA revealed an increase in p38 phosphorylation and the levels of inflammatory cytokines IL‐1α and IL‐8 in *Porphyromonas*‐positive HNSCC samples, consistent with our coculture experiments. Although previous studies have reported that Pg can activate NF‐κB signaling pathways, our coculture system did not show evidence of NF‐κB activation, and TCMA data analysis similarly showed no association between phosphorylated NF‐κB levels and the presence of *Porphyromonas*. This consistency further supports the specificity and physiological relevance of our model, suggesting that long‐term coculture with viable Pg recapitulates clinically observed signaling patterns. These results indicate that, in the context of long‐term coculture with viable Pg, p38 instead of NF‐κB may play a dominant role in mediating inflammatory responses. Previous studies have shown that p38 activation in HNSCC contributes to tumor progression by promoting angiogenesis and lymphangiogenesis through cytokine production [52, 53]. These results indicate that Pg modulates the tumor microenvironment through p38‐dependent inflammatory responses, potentially influencing the progression of HNSCC not through direct stimulation of tumor cell proliferation, but through the induction of inflammatory cytokines such as IL‐1α and IL‐8. These findings present the possibility that targeting p38 could help modulate the tumor‐associated inflammatory milieu in Pg‐positive HNSCC, although further investigation is required to clarify its therapeutic potential. Furthermore, the concordance between the clinical specimens and coculture results suggests that this culture system effectively mimics the influence of Pg in HNSCC observed in clinical specimens.

Taken together, the results indicate that the coculture model established in this study may serve as a valuable tool for investigating Pg‐HNSCC interactions, including the mechanisms of Pg colonization within tumors, Pg‐induced signaling pathways, and associated inflammatory responses. In addition, this model may be applied to screen for therapeutic agents that modulate Pg‐HNSCC interactions, suppress Pg‐induced inflammation, or inhibit Pg survival within the tumor microenvironment. Overall, this coculture system provides a powerful platform for advancing our understanding of the role of Pg in HNSCC progression.

## Conflict of interest

The authors declare no conflict of interest.

## Author contributions

YN conducted the experiments, performed data analysis, curated the data, and wrote the original draft. SO conceived and designed the project, developed the methodology, performed data analysis, curated the data, wrote the original draft, reviewed and edited the manuscript, prepared visualizations, supervised the study, managed the project, and acquired funding. MT contributed to methodology development, performed data analysis, and provided resources. SS curated the data, performed data analysis, supervised the study, validated the results, prepared visualizations, and reviewed and edited the manuscript. KI curated the data, performed data analysis, conducted investigations, managed the project, supervised the study, wrote the original draft, and reviewed and edited the manuscript.

## Data Availability

The microarray data used for the analysis of HSC‐2 cell differentiation in this study were obtained from a previously published dataset (GEO accession number: GSE97569) [35].
